# *Alu* DNA Polymorphism of Human Tissue Plasminogen Activator (*tPA*) Gene in Diabetic Jordanian Patients

**DOI:** 10.29252/ibj.23.6.423

**Published:** 2019-11

**Authors:** Salem R. Yasin, Hussam H. AlHawari, Abeer A. Alassaf, Maysa M. Khadra, Zainab A. Al-Mazaydeh, Ala'a F. Al-Emerieen, Lubna H. Tahtamouni

**Affiliations:** 1 Department of Biology and Biotechnology, Faculty of Science, the Hashemite University, Zarqa, Jordan;; 2 Department of Internal Medicine, Faculty of Medicine, University of Jordan, Amman, Jordan;; 3 Department of Pediatric, Faculty of Medicine, University of Jordan, Amman, Jordan;; 4 Department of Obstetrics and Gynecology, Faculty of Medicine, University of Jordan, Amman, Jordan;; 5 Department of Service Courses, Faculty of Science, Zarqa Private University, Zarqa, Jordan

**Keywords:** Alu, Diabetes mellitus, Polymorphism

## Abstract

**Background:**

Hypercoagulability and hypofibrinolysis are among the symptoms exhibited by diabetic patients. Our study aimed to address the polymorphic nature of *Alu* DNA fragment in the human tissue plasminogen activator gene within diabetes mellitus (DM) Jordanian patients.

**Methods:**

Genomic DNA was isolated from 76 DM patients and 60 non-diabetic Jordanian individuals, and the Alu fragment was amplified using PCR.

**Results:**

The results showed that 80% of the non-diabetic Jordanian subjects were homozygotes for the deletion of the *Alu* fragment (*Alu*^-/-^), 16.7% were homozygotes for its insertion (*Alu*^+/+^), and 3.3% were heterozygotes (*Alu*^+/-^). Besides, 36.8% of the diabetic patients exhibited the *Alu*^-/-^ or *Alu*^+/-^ genotype, and 26.3% were *Alu*^+/+^*.* The *Alu*^-/-^ genotype occurred less frequently in the diabetic individuals.

**Conclusion:**

The high frequency of the *Alu*^-/-^ genotype constitutes a protective deletion with respect to DM within the normal subjects.

## INTRODUCTION

Diabetes mellitus (DM) is a group of metabolic diseases characterized by hyperglycemia and caused by defects in insulin secretion, insulin function, or both^[^^1^^]^. Based on etiology and pathology, DM has been classified into DM type 1 (T1DM) and DM type 2 (T2DM)^[^^2^^,^^3^^]^. The major complication resulting from DM is related to the vascular system. Diabetic patients present symptoms of hypercoagulability and hypofibrinolysis; about 80% of diabetics die from thrombotic events^[^^4^^,^^5^^]^. 

The fibrinolytic system is responsible for the dissolution of fibrin blood clot^[^^6^^]^. The components of this system include tissue plasminogen activator (tPA), urokinase plasminogen activator (uPA), and inhibitors ,PA inhibitor-1 (PAI-1) and PAI-2. tPA and uPA catalyze the inactive pro-enzyme plasminogen into the dynamic plasmin. tPA is mainly involved in thrombolysis^[^^7^^-^^9^^]^. Meanwhile, tPA is synthesized in the vascular endothelial cells and is released into the blood when stimulated^[^^10^^,^^11^^]^. Its discharge, dispersion, complex formation with PAI-1 and release rate influence the tPA levels^[^^12^^-^^14^^]^. However, only tPA release rate affects the thrombolytic potential of tPA^[^^15^^]^. Hyperglycemia prevents the activity of the fibrinolytic system by stimulating the production of PAI-1. Abnormalities in the fibrinolytic system precede the development of T2DM^[^^13^^,^^16^^]^, which was associated with PAI-1 increased concentration^[^^5^^,^^17^^]^. Elevated levels of PAI-1 and tPA antigens and reduced levels of tPA have been observed in diabetes and metabolic syndrome (MetS)^[^^10^^,^^13^^]^.

The human tPA gene is located on chromosome 8p12-p11.2^[^^18^^]^, and one polymorphism, an *Alu *repeat polymorphism, has been found in intron 8 of this gene^[^^19^^,^^20^^]^. Members of the *Alu* family are short (approximately 311 base pairs) interspersed DNA elements. *tPA* polymorphism exhibits three different genotypes: *Alu*^+/+^ and *Alu*^-/-^ homozygotes and *Alu*^+/-^ heterozygote, which are based on either the insertion (I) or deletion (D) of the *Alu* element. The* Alu*^+^ allele is the derived allele^[^^21^^]^. Many populations have been reported to be dimorphic for the presence or absence of the *Alu* repeats^[^^22^^-^^25^^]^. 

There are many investigations focused on the tPA levels and its possible association with certain clinical statuses, but few have been addressed the genotypic polymorphism of the *Alu* fragment of the *tPA* gene and its effect. The *tPA* gene *Alu* polymorphism has been observed to regulate the interaction between tPA and PAI-1, and the presence of the *Alu* repeats in both alleles of this gene (*Alu*^+/+^) has been shown to associate with the elevated levels of plasma PAI-1 and tPA antigens^[^^12^^]^. Furthermore, the *Alu* polymorphism has not been found to be involved in tPA production but rather in its release rate^[^^15^^]^. Though this genotype has been implicated as a risk factor in T2DM and MetS^[^^13^^]^, an association between the I/D polymorphism and tPA synthesis or plasma levels has not been investigated yet^[^^26^^-^^28^^]^. In this study, we intended to address the genotypic and allelic distributions and possible association of the *Alu* DNA diverseness in the *tPA* gene within DM Jordanian patients.

## MATERIALS AND METHODS

**Study subjects**

In this study, 76 DM patients (26 T1DM and 50 T2DM) and 60 aged-matched healthy non-diabetic Jordanian individuals (glycated hemoglobin level [HbA1c] < 42 mmol/mol, fasting blood sugar < 100 mg/dL; data not shown) were recruited from Jordan University Hospital in Amman, Jordan from March 2016 to May 2017. Patients with impaired glucose tolerance, gestational diabetes, maturity onset diabetes of youth, or metabolic syndrome were excluded. All individuals gave their informed consents, and the study was approved by the Institutional Review Board (IRB) of the Faculty of Medicine, the University of Jordan, which conforms to the Declaration of Helsinki. 

***tPA*** **genotyping**

Peripheral blood (3 ml) was collected in EDTA tubes from each participant by venous puncture. DNA was extracted from 300 µL blood using a commercially available kit (Promega, USA). Total genomic DNA amplification was carried out as previously reported^[^^29^^]^. Briefly, 0.3 µg of genomic DNA from both normal and DM patients was subjected to amplification by PCR in a 30 µl total volume reaction containing 1 Master Mix (0.5 U of Taq DNA polymerase, 0.2 mM of dNTPs, and 1.5 mM of MgCl_2_; Promega, USA) and 0.2 µM each of 5’-flanking (GTAACCATTTAGTCCTCAGC TGTTCTCCT) and 3’-flanking (CCATGTAAGAGTA GAAGGAGACTCAGTCA) primers (the Midland Certified Reagent Co., USA). Amplification of DNA was performed in a MyCycler thermal cycler (BioRad, USA) at 96 ºC for 2 min, followed by 35 cycles of denaturation (96 ºC for 30 s), annealing at 65 ºC for 30 s, and synthesis at 72 ºC for 30 s. This process was followed by an extension step at 65 ºC for 5 min. Amplicons were electrophoresed and visualized on 2% (w/v) agarose gel with 0.5 µg/ml of ethidium bromide. Individuals carrying the *tPA* *Alu* inserts were designated homozygotes as *Alu*^+/+^ and heterozygotes as *Alu*^+/-^, as well as homozygotes for the absence of the insert as *Alu*^-/-^.

**Statistical analysis**

The observed genotypes and allele frequencies were compared with those expected in order to verify the Hardy-Weinberg equilibrium. The Chi-square test and Fisher's exact test were performed for the polymorphism frequency using Statistica software, StatSoft Inc, Tulsa, OK, USA (version 10). A value of *p* < 0.05 was considered statistically significant.

## RESULTS

**Fig. 1:**
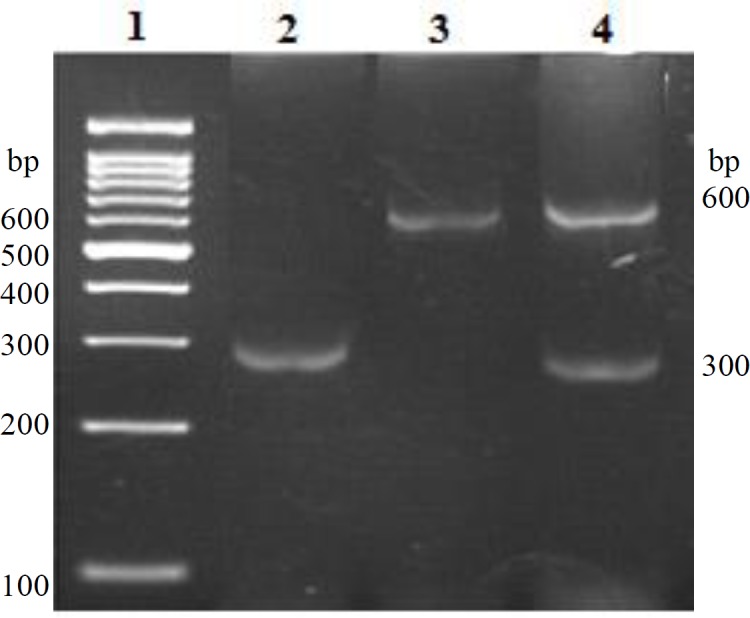
Representative *tPA* *Alu* DNA amplification. The PCR products were electrophoresed and visualized with ethidium bromide. The 300-bp band indicates the absence of the *Alu* insert, while the 600-bp band shows the presence of the *Alu* insert. Lane 1, 100 bp DNA molecular weight marker; lane 2, *Alu*^-/-^ genotype; lane 3, Alu^+/+^ genotype; lane 4, *Alu*^+/-^ genotype

DNA was successfully extracted from 60 normal non-diabetic subjects, 26 T1DM patients, and 50 T2DM patients (data not shown). The PCR amplification products of the 300 bp *Alu* region of *tPA* gene are shown in Figure 1. 

The PCR amplification results indicated that all the three genotypes, *Alu*^+/+^ (600 bp), *Alu*^+/-^ (600/300 bp), *Alu*^-/-^ (300 bp) were observed in both study groups (normal Jordanian individuals and DM patients; Fig. 1 and Table 1). The highest genotype frequency in normal non-diabetic individuals was the *Alu*^-/-^ genotype at 80.0%, while both *Alu*^-/-^ and *Alu*^+/- ^*g*enotypes were equally higher in the DM patients at 36.8% each. In DM patients, the *Alu*^+/+ ^*g*enotype percentage was 26.32%. The results in Table 1 showed a significant difference in the *Alu*^-/-^ and *Alu*^+/- ^(*p* < 0.0001) genotype distributions between the normal non-diabetic and the DM patients. Furthermore, a significant difference was demonstrated between the *Alu*^-^ and *Alu*^+ ^allelic distributions in the normal non-diabetic individuals and their DM patients' counterparts (*p *< 0.0012). On the other hand, when the diabetic patients were classified into T1DM and T2DM patients, similar genotypic and allelic distributions were noticed when they were grouped together. Table 1 shows a significant difference between normal non-diabetic individuals carrying the *Alu*^-/-^ genotype and T1DM and T2DM individuals possessing the *Alu*^-/-^ genotype (*p *< 0.0001). It has also been shown that the distribution of the *Alu*^+/-^ genotype between the three tested groups exhibited the same level of significance. Comparing the genotypic distribution of the *Alu*^+/+^ between the three experimental groups showed no significant difference. The results, assuming the recessive model (Table 1), demonstrated a significant protective effect against DM of the* Alu*^-/-^ genotype. The *Alu*^-/-^ genotype was 2.2 times more frequent in the normal non-diabetic population than in the diabetic population (odds ratio of 9.63, *p* < 0.0001).

**Table 1:** Statistical analysis of *Alu* genotypes and allelic distributions of tissue plasminogen activator (*tPA*) between normal non-diabetic (n = 60) and diabetic (DM [n = 76], T1DM [n = 26], and T2DM [n = 50]) Jordanian subjects

		**Normal group ** **% (n)**	**DM group ** **% (n)**	***p*** ** value**	**Odds ratio** **95% CI**	***p*** ** value**
**Genotype**	*Alu* ^-/-^	80.0 (48)	36.8 (28)	0.0001	Vs (*Alu*^+/+^*+ Alu*^+/-^) = 9.63 2.56-30.89	0.0001
	*Alu* ^+/-^	3.3 (2)	36.8 (28)	0.0001	Vs (*Alu*^-/-^*+ Alu*^+/+^) = 9.72 2.92-29.28	0.0001
	*Alu* ^+/+^	16.7 (10)	26.3 (20)	0.1809	Vs (*Alu*^-/-^*+ Alu*^+/-^) = 8.79 3.23-23.41	0.001
	*Alu* ^-^	0.82	0.55	0.0012		
	*Alu* ^+^	0.18	0.45	0.0012		
**Allele**		**Normal group ** **% (n)**	**T1DM group ** **% (n)**	***p*** ** < 0.05**	**Odds ratio** **95% CI**	***p*** ** value**
	*Alu* ^-/-^	80.0 (48)	34.6 (9)	0.0001	Vs (*Alu*^+/+^*+ Alu*^+/-^) = 0.02 0.001-0.29	0.0058
	*Alu* ^+/-^	3.3 (2)	38.5 (10)	0.0001	Vs (*Alu*^-/-^*+ Alu*^+/+^) = 0.02 0.001-0.29	0.0059
**Genotype**	*Alu* ^+/+^	16.7 (10)	26.9 (7)	0.2774	Vs (*Alu*^-/-^*+ Alu*^+/-^) = 0.02 0.001-0.33	0.0063
	*Alu* ^-^	0.82	0.54	0.008		
	*Alu* ^+^	0.18	0.46	0.008		
		**Normal group ** **% (n)**	**T2DM group ** **% (n)**	***p*** ** < 0.05**	**Odds ratio** **95% CI**	***p*** ** value**
	*Alu* ^-/-^	80.0 (48)	36.0 (18)	0.0001	Vs (*Alu*^+/+^*+ Alu*^+/-^) = 0.56 0.21-1.35	0.1843
**Allele**	*Alu* ^+/-^	3.3 (2)	36.0 (18)	0.0001	Vs (*Alu*^-/-^*+ Alu*^+/+^) = 0.49 0.16-1.49	0.2619
	*Alu* ^+/+^	16.7 (10)	28.0 (14)	0.1537	Vs (*Alu*^-/-^*+ Alu*^+/-^) = 0.53 0.19-1.44	0.1593
	*Alu* ^-^	0.82	0.54	0.0018		
	*Alu* ^+^	0.18	0.46	0.0018		

## DISCUSSION

The present study showed a decrease in the frequency of the *Alu*^-/- ^genotype in the diabetic patients when compared with the normal group (*p* < 0.001; Table 1). This reduction in turn might indicate that the deletion of the *Alu* fragment in the *tPA* gene has a protective role against DM. However, the relation between the polymorphic nature of* Alu* insert of the *tPA* gene and tPA enzymatic activity or its plasma levels and thus its function is still controversial. 

A number of studies have investigated the effect of circulating tPA levels on its biological activities. Works by Almer and Nilsson^[^^30^^]^ and Fuller *et al.*^[31]^ have suggested that lower tPA activity may be associated with the microthromboemobolic disease. Furthermore, it has been indicated that hypofibrinolysis due to tPA levels precedes the development of T2DM in Malaysian and north Sweden subjects^[^^13^^,^^16^^,^^17^^]^. Though the high levels of circulating tPA have been indicated, deficiency in tPA activity has been shown to correlate with several diseases such as cutaneous vasculitis^[^^32^^]^, thrombocytopenic purpura^[^^33^^]^, and diabetic retina^[^^10^^]^. Lower tPA activity was related with an increase in the PAI-1, which binds to tPA, thus reducing tPA efficiency in converting plasminogen into plasmin and, accordingly, reducing fibrin clot lysis^[^^34^^]^. Reduced fibrinolytic activity occurs in long-term diabetic patients, which could lead to the associated microthromboembolic disease^[^^10^^]^. It has long been established that T2DM is a strong cardiovascular disease risk factor^[^^35^^]^. On the other hand, the *Alu*^+/+^ genotype was associated with the elevated levels of plasma PAI-1^[^^12^^]^. Nonetheless, others did not report such an association^[^^28^^,^^36^^,^^37^^]^. 

The results of the current study demonstrated a significant protective effect against DM of the* Alu*^-/-^ genotype. However, one shortcoming of the study was the lack of measurement of plasma levels of tPA and PAI-1, as well as assaying the activity of tPA. This limitation in turn led to our inability to associate *tPA Alu* specific genotype with different risk factors of DM. Nevertheless, we believe that tPA reduced activity in patients suffering from diabetes is secondary to DM and is a risk factor for blood circularity syndromes^[^^35^^]^. However, further research is needed to investigate other effective genetic and environmental factors to find out the possible relationship between the tPA polymorphism and the onset of diabetes. 
